# Artificial intelligence based automatic quantification of epicardial adipose tissue suitable for large scale population studies

**DOI:** 10.1038/s41598-021-03150-w

**Published:** 2021-12-13

**Authors:** David Molnar, Olof Enqvist, Johannes Ulén, Måns Larsson, John Brandberg, Åse A. Johnsson, Elias Björnson, Göran Bergström, Ola Hjelmgren

**Affiliations:** 1 Department of Molecular and Clinical Medicine, Institute of Medicine, Sahlgrenska Academy, University of Gothenburg, Box 428, 40530 Gothenburg, Sweden; 2 Department of Clinical Physiology, Sahlgrenska University Hospital, Region Västra Götaland, Gothenburg, Sweden; 3 Department of Radiology, Sahlgrenska University Hospital, Region Västra Götaland, Gothenburg, Sweden; 4 Department of Signals and Systems, Chalmers University of Technology, 412 96 Gothenburg, Sweden; 5 Eigenvision AB, Bredgatan 4, 211 30 Malmö, Sweden; 6 Department of Radiology, Institute of Clinical Sciences, Sahlgrenska Academy, University of Gothenburg, Gothenburg, Sweden

**Keywords:** Biomarkers, Epidemiology, Diagnosis, Biomarkers, Cardiology, Medical research, Risk factors, Mathematics and computing, Computational models, Data processing, Databases, High-throughput screening, Machine learning, Predictive medicine, Software, Statistical methods, Circulation, Atherosclerosis, Cardiovascular biology, Cardiovascular diseases, Metabolomics

## Abstract

To develop a fully automatic model capable of reliably quantifying epicardial adipose tissue (EAT) volumes and attenuation in large scale population studies to investigate their relation to markers of cardiometabolic risk. Non-contrast cardiac CT images from the SCAPIS study were used to train and test a convolutional neural network based model to quantify EAT by: segmenting the pericardium, suppressing noise-induced artifacts in the heart chambers, and, if image sets were incomplete, imputing missing EAT volumes. The model achieved a mean Dice coefficient of 0.90 when tested against expert manual segmentations on 25 image sets. Tested on 1400 image sets, the model successfully segmented 99.4% of the cases. Automatic imputation of missing EAT volumes had an error of less than 3.1% with up to 20% of the slices in image sets missing. The most important predictors of EAT volumes were weight and waist, while EAT attenuation was predicted mainly by EAT volume. A model with excellent performance, capable of fully automatic handling of the most common challenges in large scale EAT quantification has been developed. In studies of the importance of EAT in disease development, the strong co-variation with anthropometric measures needs to be carefully considered.

## Introduction

Ischemic heart disease on the basis of coronary atherosclerosis is one of the leading causes of death in the developed world and a significant cause of morbidity. A number of well-known risk factors act systemically to promote coronary atherosclerosis^1^, but there are also local factors in the micro-environment that have atherogenic potential^2,3^, including the epicardial adipose tissue (EAT), which envelops the coronary arteries within the pericardium and has the same embryological origin as abdominal visceral adipose tissue^4^. Ever since 2003, when Mazurek showed in a landmark paper^5^ that epicardial adipocytes had an augmented inflammatory cytokine response, much research has focused on the role of excess or dysfunctional EAT as a local mediator for coronary atherosclerosis and myocardial disease. However, a prerequisite for better understanding of the role of EAT in disease is to correctly estimate its size and characteristics in large, high quality cohorts.

EAT volume (EATV) can be measured and characterized in vivo using various imaging techniques, among which computed tomography (CT) is the most frequently used, since it is readily available, fast, has a high spatial resolution and is employed clinically in coronary artery calcium scoring and/or coronary angiography^6,7^. Moreover, the attenuation of EAT in CT, or its radiodensity, quantified in Hounsfield units (HU), contains information about adipose tissue quality: increased lipid content decreases the attenuation, while fibrosis and inflammation increases the attenuation^8^. EAT attenuation on CT has been correlated with EATV^9^, but also with coronary artery disease manifestations^4,7,10,11^.

EATV can be measured accurately on CT images on condition that the pericardium defining its boundaries can be identified. Manual or semi-automatic techniques for segmentation have been widely used^7,12–15^, but are time-consuming, and the analysis of large series of cases is not feasible. In attempts to develop automatized techniques, intensity and region growing^16,17^, multi-atlas^18,19^ and deep learning^20,21^ based approaches have been evaluated. A recent systematic review of the field by Zhang et al.^22^ shows that research is trending towards the latter approach, with all of the seven methodological works on non-contrast CT images published after 2018 applying various aspects of deep learning. Five of the featured works comprise small samples, in the range of 20–53 individuals, while two works have studied larger populations, both by Commandeur et al. They describe a fully automated model based on a trained convolutional neural network (CNN)^21^, which achieved a Dice coefficient of 0.82 for EATV. This model was later adapted for multi-center studies^23^, where it was used for analysis of 776 cases and also applied in a population based study^24^ comprising 2068 individuals. A limitation of their model is that the inferior limit of their algorithm was defined by the posterior descending coronary artery, thus excluding a possibly important volume of EAT directly adjacent to the diaphragm. All CT imaging is subject to noise, which can cause both difficulties in delineating the pericardium and errors in the classification and quantification of EAT when thresholding is applied. The standard remedy for noise has been median filtering^21,23,25^. It reduces or eliminates the apparent noise in the images, but affects the whole image uniformly, which could introduce errors in the estimation of EATV. A more sophisticated approach based on a CNN trained to identify anatomical regions devoid of adipose tissue would be preferable.

The aims of the current study were; (i) to develop and test a model for fully automatic generation of high quality, complete EATV from large population based studies using non-contrast enhanced CT examinations, and (ii) to identify the most important cardiometabolic risk factors and anthropometric measures associated with EATV and EAT attenuation. To achieve these aims we used data from a subset (n = 1811) of individuals in the Swedish CArdioPulmonary bioImage Study (SCAPIS)^26^, which is a large-scale population study including CT examinations of more than 30000 individuals.

## Materials and methods

### Populations

SCAPIS is a general-population-based prospective study (www.scapis.org), to which 30154 men and women aged 50–64 years were randomly recruited from the census register at six sites (Gothenburg, Linköping, Malmö/Lund, Stockholm, Umeå, and Uppsala) between 2013 and 2018. Participants gave written informed consent and were subjected to a comprehensive examination^26^. The study is approved (# 2010-228-31M) as a multi-center study by the ethical review board in Umeå, c/o Department of Medical Research, Umeå University, 901 87 Sweden. For the present work, only subjects enrolled at the Gothenburg site (n = 6256) were included. Totally 411 randomly selected image sets were used for training and testing the software and another randomly selected 1400 image sets were used to further test the performance of the model in a larger population. To identify which factors are associated with EATV and EAT attenuation we used the test population (n = 1400, see details below).

### Study procedures and imaging

All procedures in this paper were carried out in accordance with relevant guidelines and regulations. The comprehensive study procedures in SCAPIS have been described in detail^26^. In the analyses we used data from non-contrast CT images, physical examinations and routine laboratory tests.

Briefly, all imaging in SCAPIS was performed using the same CT-scanners and protocols, Siemens Somatom Definition Flash with a Stellar detector (Siemens Healthcare, Forchheim, Germany). Care Dose 4D was used for dose optimization. Image acquisition was ECG-gated, with tube voltage of 120 kV, and refmAs of 80. The images have a matrix of 512 × 512 voxels in the axial plane, with a square DFOV in the range of 170–200 mm. All images were reconstructed using the B35f. HeartView medium CaScore algorithm, generating a slice thickness of 3 mm, with 50% overlap between slices.

### Development of CNN models and datasets used

**Figure 1:**
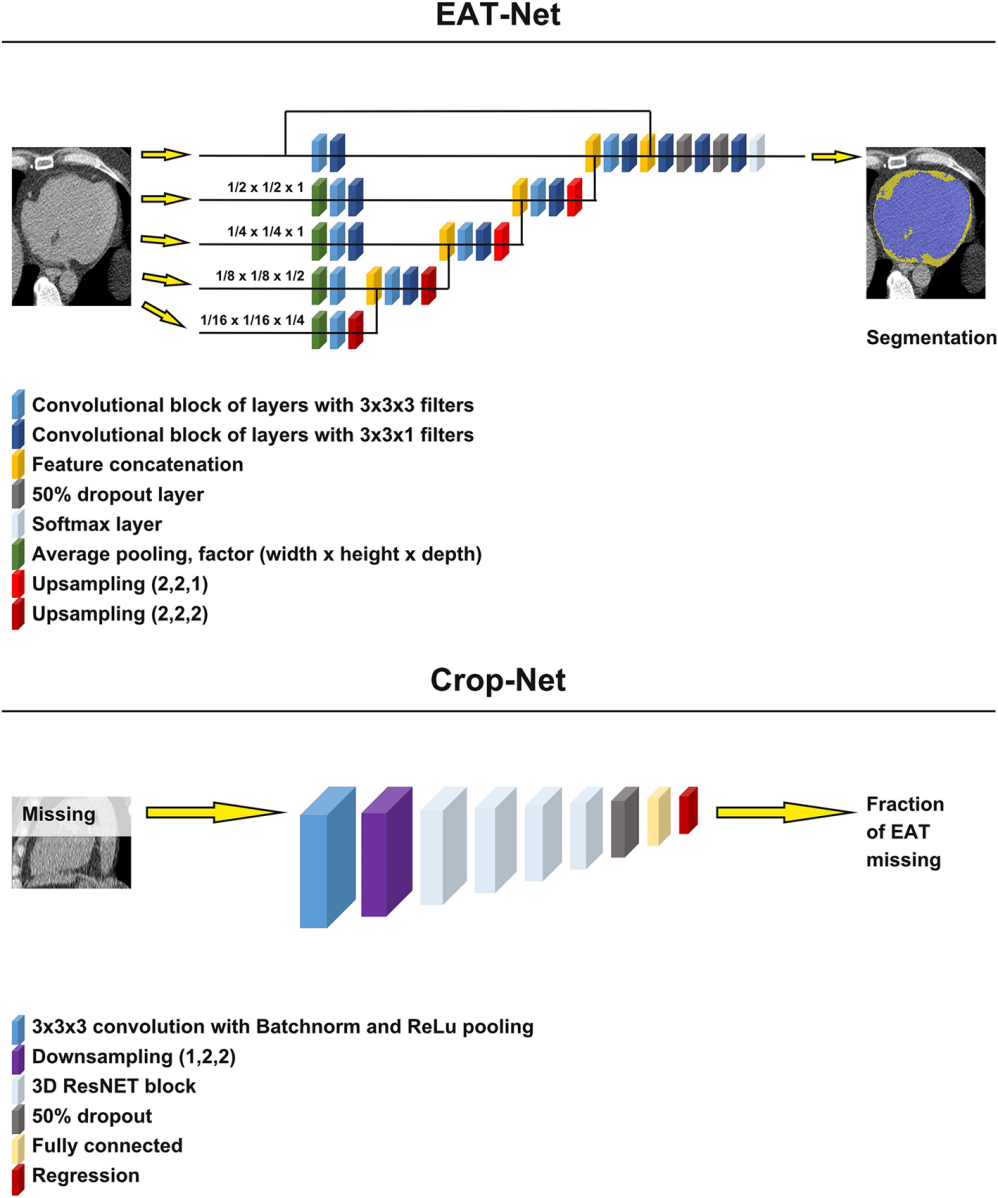
Basic design of the two CNN models which work in series. EAT-Net outputs segmentations of the heart, from which epicardial adipose tissue (EAT) volume, and EAT attenuation values are calculated. Crop-Net outputs an estimation of the fraction of EAT volume that is missing in incomplete image sets.

For the estimation of EATV we developed two CNN models which work in series. The first model, “EAT-Net” outputs a segmentation of the EAT voxels inside the pericardium, enabling calculation of EATV and EAT attenuation. The second model, “Crop-Net” estimates any missing EATV in cases where the heart is not fully represented in the image set. This problem is fairly common in the SCAPIS cohort, since the smallest possible scan volume was used in order to minimize radiation doses, increasing the risk of incomplete, cropped heart images due to patient and radiographer related issues. To develop EAT-Net, a total of 411 unique and randomly selected image sets were used (training, n = 308, validation, n = 78 and testing n = 25). In a further test of EAT-Net, another 1400 unique and randomly selected image sets were segmented by EAT-Net and visually evaluated to identify failed segmentations. To develop Crop-Net, a total of 866 image sets were selected from the dataset used for visual evaluation of EAT-Net. Crop-Net was then tested on a subset of the data used for developing EAT-Net (n = 55). The general design of the models is shown in Fig. 1.

### EAT-Net

EAT-Net is a fully convolutional neural network trained on large patches of the image and it works in a striding window fashion to segment the full image volume. Training and inference were performed using the Tensorflow^27^ and Keras^28^ frameworks. For all training steps an 80%/20% split between training and validation sets was used. All model development and hyperparameter tuning was done on the validation set and the performance of the final model was evaluated on a separate test set.

All annotations were performed by the same expert thoracic radiologist (author DM) with more than five years of experience in thoracic radiology, whose reading has previously been bench-marked against another expert reader with excellent inter-reader reproducibility (Dice coefficient for EAT = 0.9)^19^. Annotations and visual evaluation were performed in the cloud-based platform RECOMIA^29^.

When performing the annotations, a continuous line representing the pericardium was drawn in each axial slice covering the heart. The reader was free to change the window-settings, and magnification. If the pericardium was not clearly visible in parts of the actual slice, a decision was made where the pericardium was most probably located based on the neighboring slices. Training of pericardium segmentation was performed with two classes, “background” (all voxels outside pericardium) and “heart” (all voxels inside the pericardium). EAT-Net was initially trained on 29 cases where the pericardium was annotated manually in all image slices (about 70 slices per heart) by a single expert reader (author DM). The first version of EAT-Net was used to segment novel cases, which were reviewed by an expert reader (DM) and image slices with significant segmentation errors were corrected by manual annotation. The manual annotations produced in this step were used as training data in the next training session, generating a new version of EAT-Net. The process with manual correction and retraining was iterated, until the training set consisted of a total of 308 subject cases.

After finalizing training of the classes background and heart, we introduced a third class, “non-adipose tissue inside heart” to train the model to recognize areas within the pericardium certainly not containing EAT. The following procedure was used: in 30 of the training image sets, a continuous line was drawn on all slices including parts of the heart muscle and most of the heart chambers and EAT-Net was trained to segment this third class.

EAT-Net has an input size of 288 × 288 × 64 voxels and works with voxel dimensions of 0.33 × 0.33 × 1.5 mm. Data augmentation was used in all steps to generate additional training data for EAT-Net by artificially modifying the images in the following ways; (i) the HU values were varied between − 100 to + 100 (for the full patch), (ii) the patch was rotated between − 0.15 and + 0.15 radians, (iii) the patch was scaled from − 10 to + 10% in size. During training of EAT-Net, input images were randomly cropped, from any direction, to any extent, until reaching the limits of the annotated pericardium, beyond which no further cropping was done. Categorical cross entropy was used as loss function and the optimization was performed using the Adam method with Nesterov momentum.

The last part of EAT-Net is a softmax activation resulting in a score between 0 and 1 for all three classes. For each voxel the class with the highest score was chosen.

A post-processing step was applied after segmentation by EAT-Net in which the largest connected volume of the classes “heart” and “non-adipose tissue inside heart” was assumed to be the true heart. Voxels in direct contact with the index voxel, i.e. the 26 surrounding voxels in a 3 × 3 × 3 kernel, were defined as connected. Any smaller volumes of connected voxels were set to background to remove spurious voxels. The final prediction of EATV was made by selecting all voxels classified as “heart” within the Hounsfield range [− 190, − 30]. Voxels classified as “non-adipose tissue inside heart” were excluded. For segmentation using EAT-Net, no cropping was done during prediction. The entire image was processed by the network in a sliding-window manner.

### Crop-Net

Crop-Net was developed to impute missing information in image sets due to cropping, which was almost exclusively seen as incomplete representation of the heart in the superior or inferior parts of the image stacks, attributable to improper positioning or selection of scan areas during image acquisition. We hypothesized that an individual’s total EATV can be estimated from the information contained in only a few axial slices covering the center of the heart. To generate the Crop-Net training set, we selected image sets with a complete representation of all aspects of the pericardium and close to perfect segmentation (n = 866) from the dataset used for visual evaluation of EAT-Net (n = 1400). To simulate missing slices in the input CT image, a random number of image slices were then cropped away from the inferior or superior part of the image stack and Crop-Net was trained to predict the fraction of EATV that was missing.

To develop Crop-Net we used a CNN structure inspired by ResNet18^30^ but with 3D convolutions and down-sampling of layers as well as valid padding for all layers. Crop-Net takes an input of size 230 × 230 × 116 voxels and outputs a single value. For each training sample, the image stack was resampled to 0.75 × 0.75 × 1.5 mm per voxel to ensure that the entire pericardial sac fits within the input volume of the model. In addition, the examination was cropped around the pericardium with margins of (± 10, ± 10, ± 5) voxels. Finally, a random number of image slices (ranging from 0 to 50% of all slices) were cropped away randomly from either the inferior or the superior part of the heart. The fraction of EAT cropped away was calculated and used as target value for training. The same data augmentation was used as for EAT-Net.

Crop-Net was tested using a separate image dataset previously used for development of EAT-Net (n = 55), in which the whole pericardium is represented and manually annotated. To simulate a misplaced scan volume for the CT images in the test set, a fraction of the superior or inferior part of the heart was cropped.

### Evaluation of the model

The final version of the combined EAT-Net and Crop-Net model was tested in two ways; first, volume prediction was tested using 25 image sets that had manual annotation of the pericardium in all image slices as well as a complete manual annotation of “non-adipose tissue inside heart”. None of these images sets were included in the training or validation sets. Some of the image sets showed minimal superior or inferior cropping, with visually insignificant volumes of the heart not being represented in the image stack. Ground truth was defined as all voxels inside of the pericardium with Hounsfield values within [− 190, − 30] and not belonging to the class “non-adipose tissue inside heart”, when the largest connected volume filter was applied.

In addition, to identify challenges with rare anatomical variations and errors introduced from suboptimal image acquisition, 1400 randomly selected image sets were analyzed by the model and all slices of the resulting segmentations were visually assessed. The segmentation quality was scored using the following criteria:

1. Acceptable; segmentation is perfect or has only small errors, which are unlikely to affect the resulting EATV estimate. The segmentation quality of the 25 cases used for testing volume prediction was set as bench-mark for acceptable segmentations.
2. Not acceptable; significant errors in segmentation which will probably affect the resulting EATV estimate significantly. These segmentations did not fulfil the quality parameters observed in the 25 cases used for testing volume prediction.

### Published data on EATV

In order to compare our results to the literature, we tabulated a number of published studies, which have estimated EATV. Data is presented from studies, that have contributed significantly to the knowledge in the area and/or represent important cohorts and/or specific techniques for segmentation.

### What are the main predictors of EATV and EATV attenuation in a population sample?

We used data from the same cohort that was used for visual evaluation of the combined EAT-Net and Crop-Net model (n = 1400) to address how the variation in EATV and EAT attenuation could be explained by variation in different anthropometric and cardiometabolic risk factors. The total explained variance for EATV and EAT attenuation based on the following factors was analyzed using random forest regression: gender, age, weight, height, waist, hip, systolic and diastolic blood pressure, cholesterol, LDL, HDL, triglycerides, p-glucose, HbA1c, hsCRP, creatinine, active smoking, antihypertensive or cholesterol lowering medication.

### Statistics

Descriptive data was presented with percentage, median, and interquartile ranges. Model performance was evaluated using the Dice coefficient and differences in EATV between model estimates and ground truth were shown in a Bland–Altman plot. Using a random forest classifier (R-version 4.0.2, package: RandomForest), EATV and EAT attenuation was predicted from a set of 18 variables on anthropometrics and cardiometabolic risk. Total explained variance was calculated from the out-of-bag model predictions. Each variable’s percentage contribution to the explained variance was estimated using the increase in the mean squared error (MSE) of the model caused by permutation of each variable.

## Results

### Study population used for EAT-Net and Crop-Net development

There were only minimal differences in measured characteristics between the datasets used for development or testing of EAT-Net and Crop-Net (Table 1), apart from a slight female dominance in the test group of 25 individuals. The median radiation dose delivered to study participants during acquisition of non-contrast cardiac CT images was 0.28 (IQR 0.22–0.41) mSv.

**Table 1:** Characteristics of the study population.

Parameters	Datasets		
	Training and validation of EAT-Net (n = 386)	Visual evaluation of EAT-Net (n = 1400)	Testing of EAT-Net and Crop-Net combined (n = 25)
**Demographics**			
Female, n; %	187; 51.5%	719; 51.3%	14; 66%
Age in years	58 (54–62)	58 (54–61)	58 (55–60)
**Anthropometrics**			
Weight in kg	82.6 (70.4–92.7)	78.2 (68.4–89.4)	78.8 (65.2–87.9)
Height in cm	171 (164–178)	172 (165–179)	172 (166–176)
Waist circumference in cm	98 (88–106)	93 (84–101)	92 (76–104)
Hip circumference in cm	103 (98–109)	102 (97–107)	103 (94–109)
**Cardiometabolic risk factors**			
Current smoking, n; %	60; 15.5%	151; 10.8%	5; 20%
Systolic blood pressure in mm Hg	123 (114–135)	120 (110–132)	118 (114–128)
Diastolic blood pressure in mm Hg	75 (69–81)	72 (66–80)	72 (67–79)
Antihypertensive medication, n; %	92; 23.8%	245; 17.5%	5; 20%
Cholesterol in mmol/l	5.7 (5–6.4)	5.5 (4.9–6.2)	5.6 (5.1–6.4)
LDL in mmol/l	3.8 (3.0–4.4)	3.6 (3.1–4.3)	3.6 (2.9–4.5)
HDL in mmol/l	1.6 (1.3–2)	1.6 (1.3–2)	1.8 (1.5–2.2)
Triglycerides in mmol/l	1.1 (0.81–1.6)	1 (0.76–1.5)	1.1 (0.73–1.6)
Cholesterol lowering medication (n; %)	92; 23.8%	96; 6.9%	2; 8%
Blood glucose in mmol/l	5.7 (5.3–6.1)	5.5 (5.2–5.9)	5.7 (5.3–5.9)
HbA1c in mmol/mol	35 (33–38)	34.5 (32.8–37)	35 (33–37)
hsCRP in mmol/l	1.4 (0.66–2.8)	0.97 (0.54–2)	0.98 (0.54–2.8)
Creatinine in mmol/l	79 (69–88)	78 (69–88)	74 (68–83)

Continuous numerical variables are represented with their median and, in brackets, interquartile range.

### Evaluation of combined EAT-Net and Crop-Net

**Figure 2:**
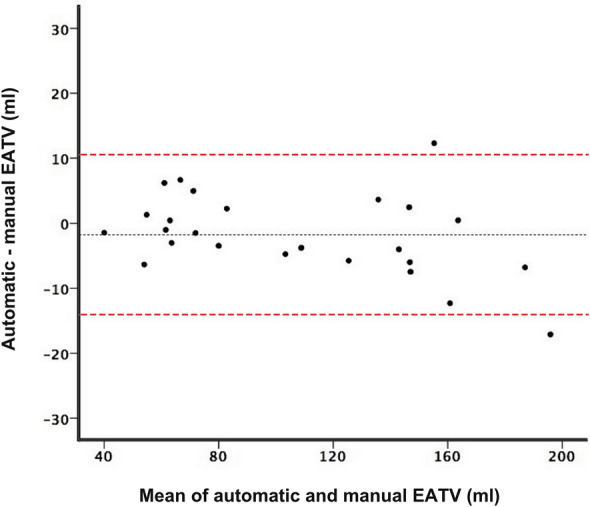
Bland–Altman plot showing the agreement between epicardial adipose tissue volumes (EATV) derived from automatic segmentations by the software and manual ground truth. The black dotted line represents the mean difference between the methods (− 1.76 ml), and the red dashed lines represent the limits of agreement.

The mean Dice coefficient was 0.90 in the 25 test cases, when total EATV quantified by the combined model was compared to manually segmented ground truth EATV (Table 2). EATV quantified by the combined model was at an average 1.76 ml smaller than ground truth. The Bland–Altman plot shows (Fig. 2) that the combined model tends to slightly underestimate the EATV and has a tendency to perform better at lower volumes.

**Table 2:** Individual results on epicardial adipose tissue (EAT) volume from the 25 test cases.

Case no	Automatically segmented total EAT volume (ml)	Manual ground truth total EAT volume (ml)	Absolute error (ml)	Relative error (%)	Dice coefficient of total EAT volume
1	163.89	163.43	0.45	0.28	0.91
2	83.93	81.7	2.24	2.74	0.90
3	73.61	68.63	4.97	7.25	0.87
4	137.62	133.96	3.65	2.73	0.89
5	100.93	105.66	4.73	4.48	0.92
6	154.64	166.96	12.32	7.38	0.90
7	183.60	190.39	6.79	3.57	0.89
8	143.27	150.75	7.48	4.96	0.88
9	140.96	144.97	4.01	2.77	0.89
10	122.55	128.31	5.76	4.49	0.93
11	187.32	204.43	17.11	8.37	0.90
12	39.34	40.79	1.45	3.55	0.91
13	71.12	72.6	1.48	2.04	0.89
14	147.78	145.32	2.46	1.69	0.89
15	143.78	149.78	5.99	4.00	0.88
16	78.26	81.73	3.48	4.25	0.94
17	62.00	65.03	3.03	4.66	0.92
18	55.46	54.16	1.30	2.40	0.90
19	69.95	63.28	6.67	10.5	0.87
20	60.99	61.99	1.01	1.63	0.93
21	50.78	57.13	6.35	11.1	0.89
22	64.12	57.92	6.19	10.7	0.88
23	63.15	62.71	0.44	0.70	0.88
24	161.46	149.14	12.32	8.26	0.86
25	106.97	110.75	3.78	3.41	0.92
Mean	106.70	108.46	5.02	4.72	0.90

EAT volumes automatically segmented by the model are compared to manually segmented ground truth EAT volumes for each case, showing the absolute and relative errors and the Dice coefficient.

**Figure 3:**
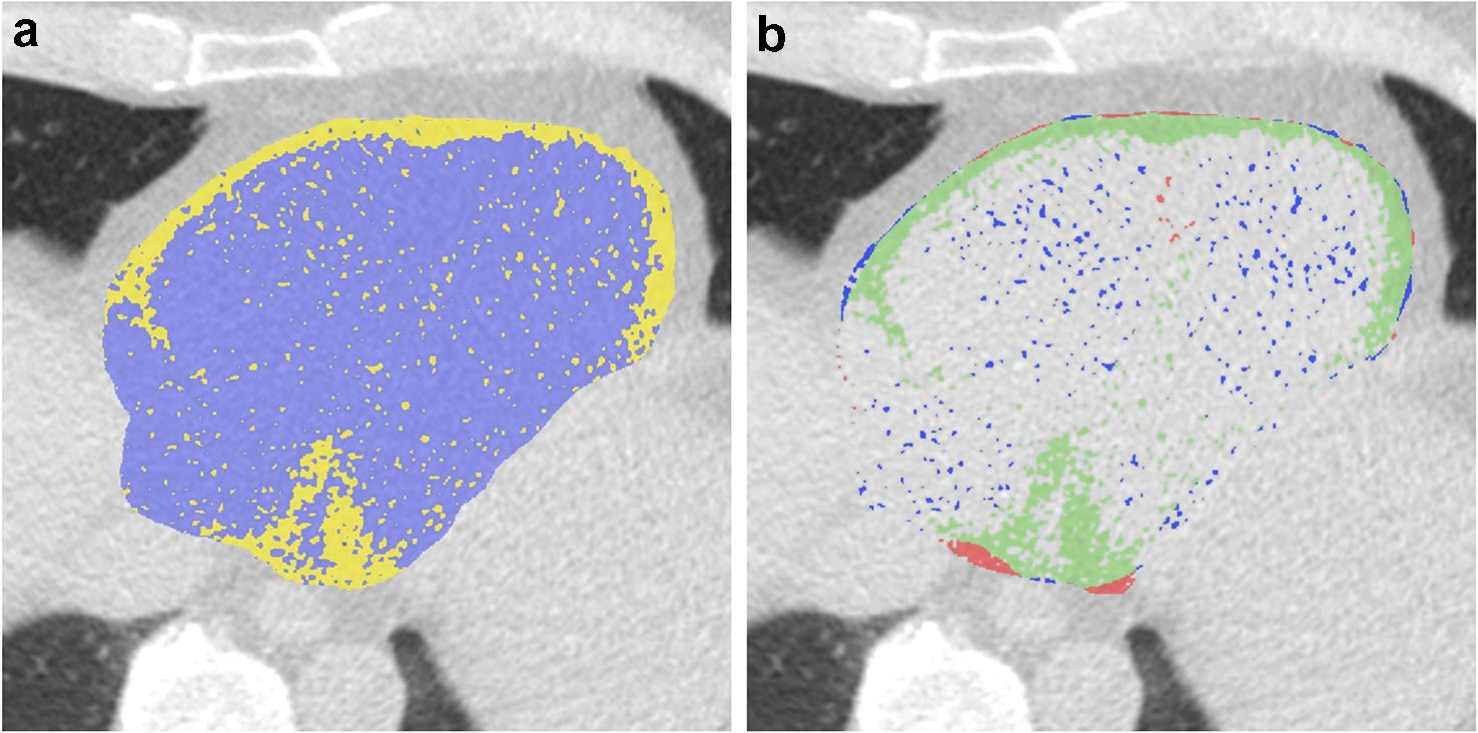
The effect of image noise on voxel classification. (**a**) An axial slice after application of the largest connected volume filter and thresholding (− 190 to − 30 HU) but prior to application of the anatomical noise filter. Epicardial adipose tissue (EAT) is represented in yellow. It is evident that the chambers of the heart contain a number of voxels wrongly classified as EAT. (**b**) The same axial slice, but after also applying the anatomical noise-filter. Here true EAT is represented in green, while voxels removed by the filter are blue. Voxels in red are not affected by the filter. It can be seen that most of the voxels misclassified by thresholding are correctly identified by the filter.

Visually, significant amounts of false positive voxels in the chambers of the heart could be seen when classifying “heart” voxels based solely on the Hounsfield value threshold for adipose tissue [− 190, − 30] (Fig. 3). The problem ranged from random voxels, to confluent clusters, and showed large variation between individual image sets. Our anatomical noise suppression performed well on visual assessment and also reduced the average error in the model’s estimation of ground truth EATV with 11.1%, from a relative error of 5.31% (SD: 3.92) to 4.72% (SD: 3.12).

### Visual evaluation of combined EAT-Net and Crop-Net

**Figure 4:**
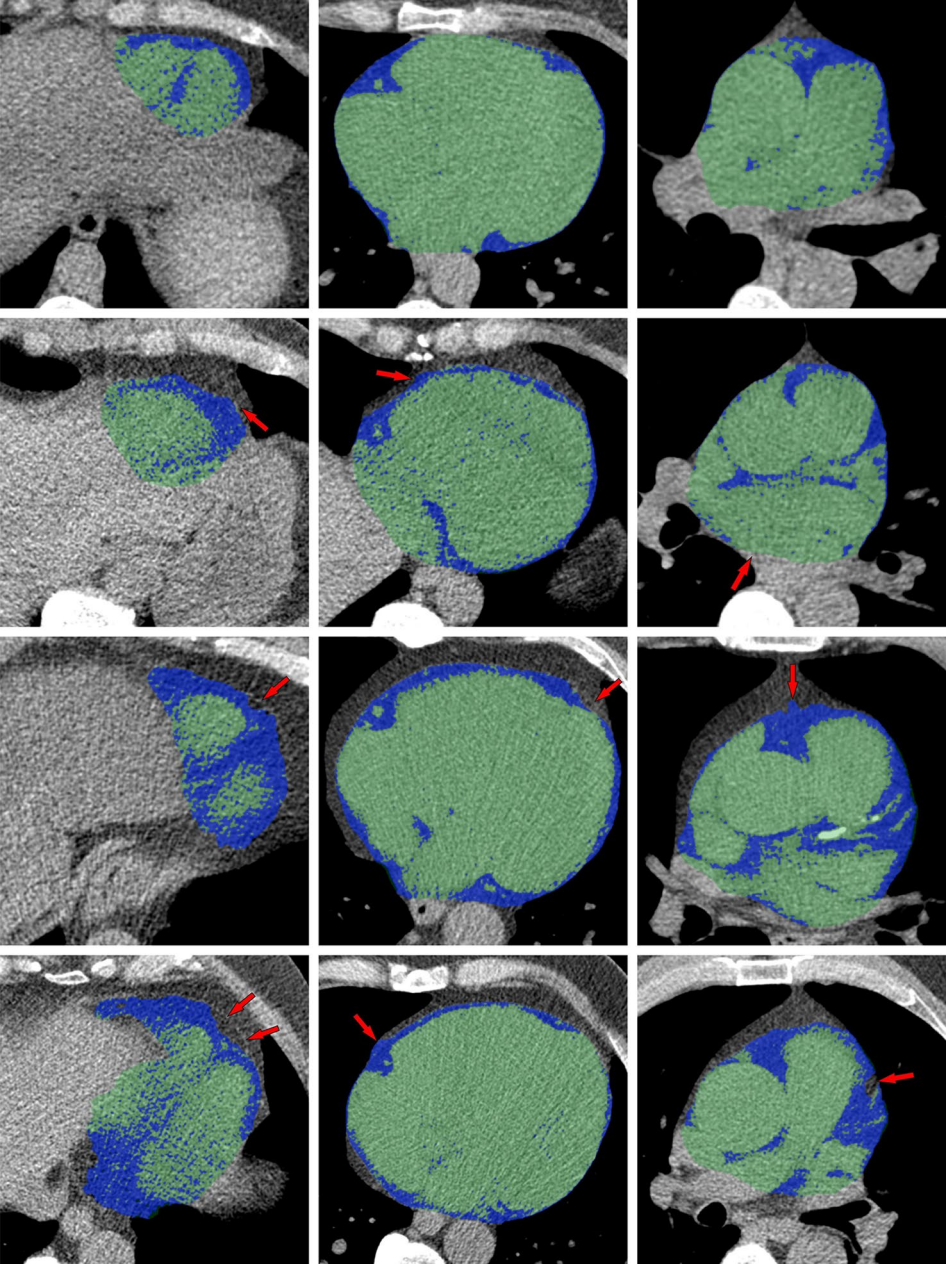
Examples of automatic segmentations. Epicardial adipose tissue (EAT) is colored blue, non-EAT within the pericardium is colored green. Each row, from left to right, represents an inferior, mid- and superior axial slice from the same individual. Imperfections in the automatic segmentations are noted with red arrows. As we can see, the last case shows some slight areas of missing EAT in the most inferior and superior parts respectively. At whole-heart level, when measuring the total EATV, these errors were found to be insignificant.

**Figure 5:**
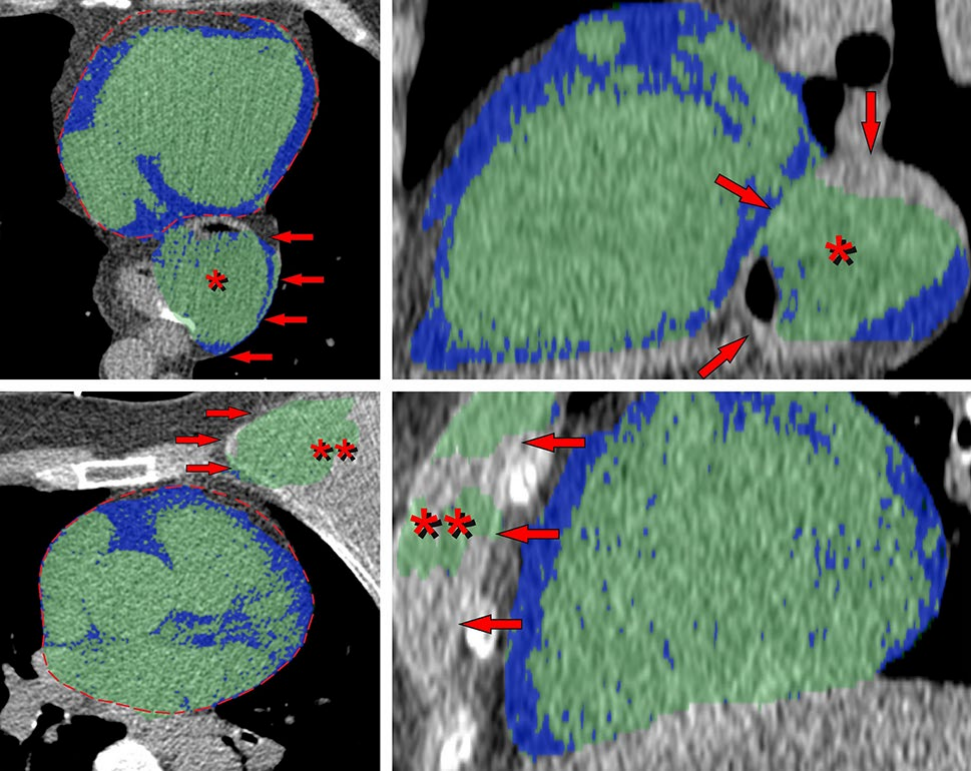
Examples of failed segmentations found in the 1400 cases that were visually evaluated. Epicardial adipose tissue (EAT) is colored blue, non-EAT within the pericardium is colored green. The most significant segmentation errors are marked with red arrows. The correct pericardial contour is marked with a dashed red line. Each row, from left to right, represents a mid-axial and a mid-sagittal (side-view) slice from the same individual. A large hiatal hernia located posterior to the heart, the probable reason for failure in the first of the two cases, is marked with an asterisk (*). In the second case the reason for segmentation failure seems to be a left-sided breast implant, marked with two asterisks (**).

When a total of 1400 examinations were analyzed using the final model, the absolute majority (99.4%) showed acceptable segmentations (Fig. 4) within the range of the 25 cases used for testing volume segmentation, implying a maximal deviation of 12% from ground truth EAT volumes. Only eight image sets (0.6%) showed unsatisfactory segmentations (Fig. 5). In four of these we found major anatomic variations: a large hiatal hernia, breast implants, a reconstructed esophagus after surgery, and left sided diaphragmal paresis respectively. The resulting median EATV from these eight failed segmentations was 112 ml (range 50–172 ml). In a linear regression analysis using weight and waist to predict EATV, these eight volumes could not be identified as outliers, all being within 1.7 standard deviations of predicted values.

### Evaluation of Crop-Net

Cropping that potentially affected EATV estimates was mainly seen in the superior (18% of image sets) and inferior (12% of image sets) parts of the image stack, while cropping in other orientations was rare (< 1%). Cropping was usually minor, corresponding to a few image slices missing, but in some cases substantial, with between an estimated 10–30% of the image slices missing (> 10% cropping was seen superiorly in 3.6% and inferiorly in 5.8% of image sets). Results from testing of Crop-Net showed that true EATV could be predicted within a 6% margin when as much as 40% of the heart was missing in either the inferior or superior portion of the image stack (Table 3). The correlation between predicted and true missing EATV was similar for the superior and inferior parts of the heart, with linear regression coefficients of 0.954 and 0.953 respectively (p < 0.001).

**Table 3:** Performance of Crop-Net in imputing missing image information with different fractions of the image stack missing.

Missing fraction (%)	Image incomplete superiorly		Image incomplete inferiorly	
	Mean absolute error (ml)	Mean relative error (%)	Mean absolute error (ml)	Mean relative error (%)
0–10	0.979	0.855	0.308	0.346
10–20	3.57	3.13	3.29	2.96
20–30	4.22	3.61	4.61	4.25
30–40	4.34	3.71	5.03	4.82
40–50	6.22	5.26	5.57	5.26

Results from when the upper (left columns) and the lower (right columns) part of the heart have missing slices are shown. EAT volumes of the test cases were in the range of 33.1–278 ml, with a mean of 112 ml.

### Published data on EATV

Ten reports on various cohorts (Table 4), totally including slightly more than 14,000 individuals, when adjusting for some overlap between cohorts^23,24^ show large variations in estimates of mean (or median) EATV, which ranges from 73 to 159 cm^3^. The weighted average EATV for all cohorts is 95.5 ml. The majority of works employ semi-automated methods for EAT quantification (weighted average 99.1 ml), whereas the two publications relying on automated quantification show among the lowest amounts of EATV. One publication on necropsy material^31^ containing no other adipose tissue than EAT shows the lowest EATV, slightly below 70 ml, when male and female subjects are pooled.

**Table 4:** Characteristics of a selection of studies presenting data on epicardial adipose tissue volumes (EATV) derived from mostly non-contrast CT scans.

Reference	Cases (n)	Study population	Segmentation technique used and its reference	BMI/Waist	EATV in ml (mean or median*)
Ding et al.^42^	1119	MESA, population sample	Semi-automatic, measures a subsection of pericardial fat, method described in Wheeler et al.^35^	28/99	82*
Britton et al.^34^	3086	Framingham, population sample	Semi-automatic, method described in Rosito et al.^33^	27.7/97	111 ± 43
Mahabadi et al.^32^	4093	HNR, population sample	Semi-automatic, method described in Mahabadi et al.^15^	–/94	86*
Forouzandeh et al.^14^	760	CACS screening in population at risk	Semi-automatic, original method description	31/–	127 ± 61
Kunita et al.^6^	722	CACS screening in population at risk	Semi-automatic, method described in Sarin et al.^36^	24/88	107*
Hindsø et al.^31^	132	Forensic autopsy	Semi-automatic segmentation of EATV after removal of extra-pericardial fat	25/–	73.0 (m)
				26/–	64.8 (w)
Commandeur et al.^23^	776	EISNER n = 638, LDL n = 79, CACS screening Erlangen n = 23, Seoul n = 36	Non-contrast, CNN, original model developed in Commandeur et al.^21^		86.75 (64.23–119.61)
Marwan et al.^12^	237	Clinical data	Semi-automatic, original method description	27/–	159 ± 76
Mancio et al.^13^	574	EPICHEART, patients with aortic stenosis and CAD	Semi-automatic, original method description	28/–	109.7 ± 55.9
Eisenberg et al.^24^	2068	EISNER trial, asymptomatic	Fully automatic, method described in Commandeur et al.^21,23^	27/–	78.5 (55.9–106.0)
Milanese et al.^11^	1344	ALTER-BIO, clinical indication for CCTA	Contrast enhanced, semi-automatic method described in Milanese et al.^7^		90.52 (11.27–442.21)

### Main predictors of EATV and EAT attenuation

**Figure 6:**
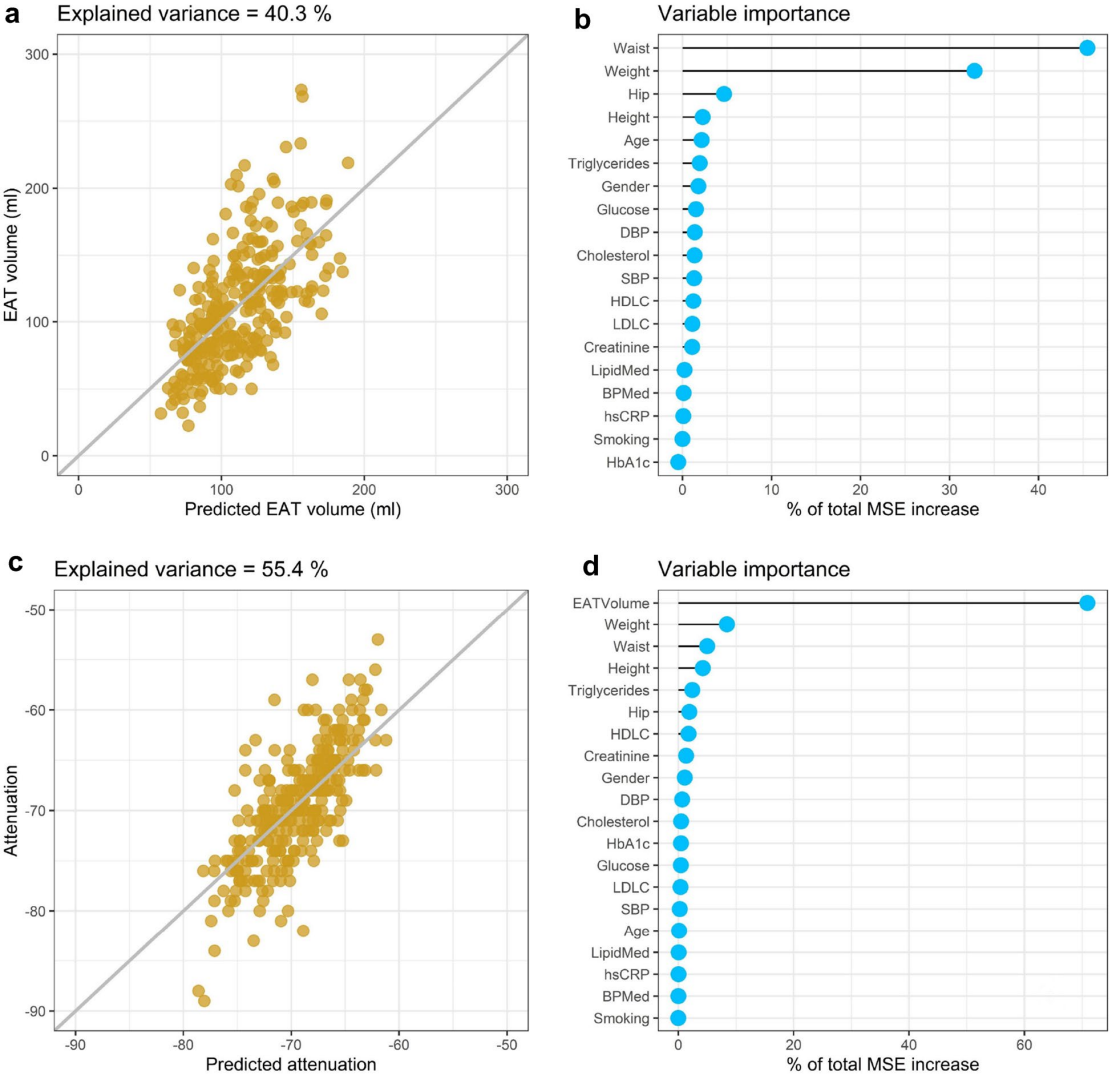
Epicardial adipose tissue (EAT) volume and attenuation and their association with anthropometrics and cardiometabolic risk factors. Using a random forest classifier, EAT volume (**a**) and attenuation (**c**) was predicted from a set of 18 anthropometric measures and cardiometabolic risk factors. Total explained variance was calculated from the model's prediction. Variable importance (**b** and **d**) was calculated from the increase in the mean squared error caused by permutation of each variable.

Anthropometry and cardiometabolic risk factors could together explain at total of 40.3% of the variation in EATV (Fig. 6a). When the individual factors were tested in a variable importance analysis, it was clear that anthropometry (weight and waist) were the most important predictors, together explaining up to 78.3% of the increase in mean squared error (MSE) of the regression model (Fig. 6b).

Anthropometry and cardiometabolic risk factors could together explain at total of 55.4% of the variation in EAT attenuation (Fig. 6c). EATV was the most important predictor of EAT attenuation, explaining more than 70.9% of the increase in MSE (Fig. 6d). In a model, where EATV was not included, 22.5% of the variation in EAT attenuation was explained by anthropometry and cardiometabolic risk factors, among which weight and waist were the most important predictors (together explaining 42.6% of the increase in MSE).

## Discussion

In this report we have presented a model for fully automatic segmentation of EAT volumes in non-contrast enhanced cardiac CT suitable for unsupervised use in large scale studies. The model is specifically adapted to cope with problems of noisy and/or incomplete image sets, which are likely to be encountered in large population studies. The model consists of two CNNs in series, trained using supervised learning based on a comprehensive set of detailed manual annotations.

When tested on 25 expert manual segmentations, the model had an average Dice coefficient of 0.90. It was capable of delivering acceptable segmentations in 99.4% of 1400 visually evaluated image sets. Effects of noise induced artifacts on EATV quantification could be reduced by up to 11% with the model’s anatomically trained noise suppression. The model showed its ability to predict correct total EAT volumes on incomplete image sets with a precision of over 95%. Finally, we showed that 40% and 55% of the variation in EAT volume and attenuation respectively could be explained by anthropometrics and cardiometabolic risk factors.

### Performance of the model

Most previously published data from larger cohorts relies on measurements with manual annotations or semi-automatic techniques^6,7,13,32–36^. In a recent systematic review by Zhang et al.^22^, seven studies reporting on model-based methods, all prior to 2017, and nine studies reporting on deep-learning based methods applied to non-contrast cardiac CT were included. Among these, the fully automatic model described by Commandeur et al. is the only one developed and tested in a larger population sample, with an impressive Dice coefficient of 0.82 for EATV in the first report^21^, which was later improved to 0.87, when the model was adapted to multicenter use^23^. Our model performed well in comparison, with a Dice coefficient of 0.90, when tested against expert measurements in 25 fully manually segmented image sets. When developing models intended for fully automatic use in large cohort studies, it is important to test model performance in large series of data. This can identify segmentation problems related to e.g. anatomical variations. To validate the performance of the model in large-scale fully automatic use, 1400 unique image sets were analyzed. Visual assessment showed consistent segmentation quality, with only eight image sets (0.6%) deemed unacceptable due to large segmentation errors. The failed segmentations were not easily identified from the generated data, since their EAT volumes were well within the range of EAT volumes seen in acceptable segmentations. To avoid manual quality control of large series of data, future work could use information on geometrical distribution of the segmentation or ratios between outer surface area and volume to automatically identify these cases.

Large population studies using CT imaging by necessity strive to reduce radiation doses, which results in both a low signal-to-noise ratio and a risk of generating incomplete image sets, since the smallest feasible scan volumes are used. The error caused by image noise was reduced by training the model to identify areas within the images which are certainly devoid of EAT. This training reduced the relative error in estimation of EAT volumes by 11% Incomplete image sets were common in the dataset, but were efficiently handled by Crop-Net, which imputed missing EAT volumes with good precision, estimating total volumes within 95% of ground truth.

### EAT volumes in relation to previously published data

The average EATV in the current study was 113 ml (range 22–320 ml), which is well in line with many previously published estimates of EATV, although the variation between studies is substantial and not always consistent with variations in other measures of anthropometrics. Eisenberg used an automated segmentation technique similar to ours^21,24^ in 2086 cases reporting an average EATV of 78.5 ml, even though the size of cohorts, weight and BMI are comparable. This exemplifies the difficulties in establishing common reference values in the area. Differences between our estimates and those of Eisenberg are quite large and probably not fully explained by the omission of the most inferior part of the epicardial tissue in their work. An interesting work by Hindsø^31^ reports EATV from forensic autopsy material and again shows substantially lower EATV than in our current report, despite similar weight and BMI. The degree to which post mortem data on EATV can be compared to in vivo data is not known. However, their work is important in the sense, that it reflects anatomically perfectly segmented EAT. Published data relying on semi-automatic measurements show varying EATV, the study by Britton et al.^34^ reporting volumes similar to ours, Mahabadi^32^ reporting lower volumes and Marwan^12^ substantially higher volumes, despite comparable anthropometric data. Reasons for the discrepancies could be several, including different segmentation techniques, effects of noise on thresholding, or true biological variation, although the latter seems less plausible given the relatively large cohorts. Unfortunately, this field of research does not yet have any standardized normal values for EATV.

### EAT volumes versus EAT attenuation

It is challenging to single out the independent effect of EATV on disease in cross-sectional studies, since EATV changes in parallel with other anthropometric measures and especially with other estimates of ectopic accumulation of adipose tissue^35,37^. Our cross-sectional analyses show that as much as 50% of the variation in EATV is explained by variations in anthropometric measures (weight and waist). It is not known how EATV is regulated, or if it changes in parallel with other fat depots under interventions. A few select, small longitudinal studies seem to show that EATV changes with body weight and also appears to increase with age^10,37^. To advance our knowledge further, more high-quality data is needed on serial measurements of different fat depots under interventions that affect weight.

The attenuation, or radiodensity of EAT has been proposed as a possible independent factor in cardiovascular risk assessment^4,38^. Our data shows that there is a strong inverse association between increased EAT volume and attenuation, i.e. large EAT depots have lower radiodensity. This may be explained by a relative increase in tissue lipid content from adipocyte hypertrophy^8,39^. In a study by Franssens et al.^40^ including 140 individuals, the difference in EAT attenuation between patients in different risk strata was very small, ranging between 1 and 4 HU, while a study by Mahabadi et al.^41^ including 94 individuals showed a difference in EAT attenuation of 2.2 HU between patients with and without previous myocardial infarction. In our material, noise affected the estimation of EAT volume, which raises questions about the reliability of attenuation measurements. Given the small reported differences in EAT attenuation, minor artifacts from noise may be an important source of error. In future studies of EAT attenuation, the strong co-linearity with EAT volume and the effects of noise need to be taken into account.

### Limitations

The requirements to minimize radiation exposure of the participants carry two major limitations: (a) there is a significant share of incomplete images in our material, since scan areas were the smallest possible with reluctance to repeat scans, and (b) images have higher noise levels than standard clinical images. Incomplete images would, if left unaddressed, lead to varying degree of error in the estimation of EATV, or rather, the exclusion of a significant amount of cases in a population study, reducing statistical power. Noise, on the other hand, influences the estimation of EATV through misclassification of voxels, potentially affecting any image set in part or entirely. To cope with these challenges, we needed to expand the training of the model to incorporate a dedicated anatomically based noise suppression solution, and also a solution for cases with incomplete representation of the heart. The final model reduced the error attributable to noise and was able to impute missing EATV in incomplete images with high precision.

All manual segmentations were produced by the same expert reader in this work, which could be considered a shortcoming, but also has some advantages. Firstly, in a previous work^19^, our reader was compared to another expert reader with more than 10 years of experience with cardiac CT. The mean Dice coefficient for segmentations of the two readers was 0.90 and the linear regression coefficient was 0.96, well in line with the work of others^23,42^. Secondly, we believe that much of the inter-reader variability can be accounted for by difficulties in placing the annotation line exactly on the very thin pericardium, which especially in non-contrast images is not always clearly visible. Using ground truth from several readers poses a risk of introducing uncertainty in the training steps of a model and may negatively affect its final performance.

The use of active learning in the training process could potentially introduce some degree of bias in the final model since training data no longer strictly follows the population distribution. Despite this, our model performed well on the randomly selected test data.

K-fold cross-validation might have been used to increase generalizability, given that the initial training set was relatively small, but we believe that the currently used method takes better advantage of our active learning process.

In our test of 1400 cases our model showed failed segmentations in 0.6% of the cases. These eight cases had EATV estimates within the normal range. The current model cannot find them automatically, and this is a limitation. Future work could use information on geometrical distribution of the segmentation to automatically identify these cases.

Finally, our model has not been tested on an external dataset. However, the model is carefully designed to handle image datasets from the SCAPIS study, with a final goal to segment EATV in all available SCAPIS image data.

### Future directions and impact on clinical decision making

The current model could lay the foundation for future development of clinical risk prediction models based on EATV and EAT attenuation measurements. However, this will have to await future studies, applying the model to large population data. In the near future, the model will be applied to the SCAPIS study, containing population data from 30,000 individuals. The association between EATV and EAT attenuation and coronary artery disease markers, atrial fibrillation and heart failure will be investigated. Also, we will use the model in a smaller study (about 2000 individuals with varying degrees of glucose metabolism disorders ranging from normal over impaired glucose tolerance to fulminant type-2 diabetes) focused on the association between EATV and diabetes.

## Conclusion

A model with excellent performance was developed, capable of fully automatic quantification of EAT volumes and attenuation in non-contrast cardiac CT images, with only a small fraction of analyzed cases (0.6%) being considered failed segmentations. The model’s abilities to cope with noise-induced artifacts and incomplete image sets should be of great value in large-scale studies. Performance metrics of the model are well in line with those of previously published models, and measured EAT volumes compare well with previously reported data. We could demonstrate a strong co-linearity between EAT volume, EAT attenuation and anthropometric measures, something which needs to be addressed in future studies on the role of EAT in cardiovascular disease.
